# Assessment of Relative Energy Deficiency in Sport (REDs) Risk among Adolescent Acrobatic Gymnasts

**DOI:** 10.3390/jpm14040363

**Published:** 2024-03-29

**Authors:** Omri Besor, Noam Redlich, Naama Constantini, Michal Weiler-Sagie, Efrat Monsonego Ornan, Shira Lieberman, Lea Bentur, Ronen Bar-Yoseph

**Affiliations:** 1 Department of Family Medicine, Maccabi Healthcare Services, Tel Aviv 6812509, Israel; 2 Heidi Rothberg Sports Medicine Center, Shaare Zedek Medical Center, Faculty of Medicine, The Hebrew University, Jerusalem 9103102, Israel; 3 Institute of Biochemistry and Nutrition, The Robert H. Smith Faculty of Agriculture, Food and Environment, The Hebrew University, Rehovot 7610001, Israel; 4 Rappaport Faculty of Medicine, Technion-Israel Institute of Technology, Haifa 3109601, Israel; 5 Department of Nuclear Medicine, Rambam Health Care Campus, Haifa 3109601, Israel; 6 Ruth Rappaport Children’s Hospital, Rambam Health Care Campus, Haifa 3109601, Israel

**Keywords:** relative energy deficiency in sport, bone density, acrobatics, young athletes

## Abstract

Energy imbalance exposes athletes to relative energy deficiency in sports (REDs) syndrome. Data on energy consumption, REDs, and bone mineral density (BMD) in adolescent acrobatic gymnasts, especially in males, are scarce. Our aim was to examine the eating habits, energy balance, body composition, and BMD of these athletes. In this study, 18 healthy adolescents participating in competitive acrobatic gymnastics completed a questionnaire, underwent a dual-energy X-ray absorptiometry scan (DXA), received a food log, and had their activities monitored for 3 days. Eighteen acrobats were enrolled (mean age: 14.3 ± 1.2 years; males: 6/18). The mean total body BMD Z-score was 0.4 ± 1.0. Top-position acrobats (7/18) had significantly lower total body BMD Z-scores than base-positioned acrobats (−0.2 ± 0.3 vs. 0.8 ± 0.3, *p* = 0.032), though their forearms were not significantly different (0.2 ± 0.5 vs. 0.8 ± 0.7, *p* = 0.331). No sex differences were found for BMD Z-scores, BMI, or energy availability. The BMD parameters of the acrobats were within the normal range for a healthy pediatric population, although three had low BMDs (<−1 SD) for healthy athletes. Total body and LS BMD Z-scores were significantly lower in top-position athletes compared to base-position athletes. These findings suggest personalized (top vs. base) training programs (high-impact training) that may achieve better health outcomes.

## 1. Introduction

Athletes are required to consume adequate energy, especially during periods of intense training, to maintain optimal body composition and health and maximize training effects. 

An imbalance between energy consumption and expenditure can cause low energy availability (LEA) that could result in relative energy deficiency in sport (REDs) [1,2,3,4,5,6]. 

REDs negatively affects athletic health and performance, as [1] detailed in the latest IOC (International Olympic Committee) consensus statement (Table 1) [2]. LEA for longer time periods is correlated with worse health and performance outcomes [2]. Adolescent athletes with REDs may experience a delay in growth and development, with a risk of compromising peak height and bone mass [7]; a loss of muscle mass; an increased risk of fatigue, depression, injury, and illness; decreases in the response to training, coordination, concentration, and muscle strength; and a prolonged recovery process [8]. Underlying causes and symptoms differ for males [9] and females [10,11]. For example, in females, REDs may delay the age of menarche and cause menstrual dysfunction [12,13], thus increasing the risk of reduced bone density, which can be irreversible even if menstruation returns to normal [12,14]. REDs can decrease the response to training, coordination, concentration, and muscle strength, as well as cause depression, glycogen store depletion, and an increased tendency toward injury [1,15,16]. Athletes in sports that require a lean physique (either for performance or esthetics) are prone to developing these negative outcomes.

Acrobatic gymnastics is a young, non-Olympic, fast-growing competitive discipline within gymnastics that includes long-duration training and a focus on body image. Teams of two to four gymnasts work together to perform figures consisting of acrobatic moves, dancing, and tumbling, all set to music. Training involves individual elements, group activities (throwing, catching, pyramids, and lifting), and choreographics (jumping and twirling) [17]. Positions are clearly defined within each team. “Base” position athletes are typically older and stronger. “Top” position athletes are usually younger and lighter [1,6].

To the best of our knowledge, studies focusing on EA, bone mineral density (BMD) and REDs implications in adolescent acrobatic gymnasts are yet to be reported, although it is suspected that the sport has a high REDs prevalence.

Our study’s aim was to complete a comprehensive analysis of the eating habits, energy balance, body composition, and bone density of adolescent acrobatic gymnasts using questionnaires, an activity tracker, and a body composition assessment.

This study’s protocol is detailed in Section 2, followed by Section 3, which reports the main outcomes of the study (including figures and tables). In Section 6, the results are discussed in view of the known literature.

## 2. Materials and Methods

### 2.1. Study Design

A clinical prospective cohort study was carried out between the years 2020 and 2021 in a single university-affiliated tertiary medical center. Twenty acrobatic gymnasts from the northern part of Israel who fulfilled the inclusion criteria (see below) were invited to participate in the study. Two participants withdrew from the study due to scheduling difficulties. All enrolled participants were invited for a single visit that was completed during the active training season (between competitions) The study assessments were carried out on Fridays for the most part (a shorter school day). The inclusion criteria were adolescents (ages 12–18) engaging in competitive acrobatics for at least 10 h per week for at least one year. Excluded were those involved in irregular training, recovering from an injury incurred in the previous month, with a chronic medical condition, or taking steroids or some other medication that might impact bone health. This study was conducted in accordance with the amended Declaration of Helsinki. A local institutional review board approved this protocol (RMB-0170-21). Since the individuals studied were minors, the participants signed assent forms, and their parents signed consent forms.

Of the 18 gymnasts who signed the consent form, 66% were females (12/18). The mean age was 14.3 ± 1.2 years for females and 14.3 ± 2.5 for males. Six females were pre-menarche (age range: 12.8–14.2 years). None of the female participants reported the use of contraceptives. Half of the gymnasts were competitive on an international level (seven females and two males; mean age: 15.0 ± 2.0 years); the others were competitive on a national level (five females and four males; mean age: 13.7 ± 1.1 years). The international group trained 6.7 h more each week (a total mean of 25.5 ± 4.34) than the national group (mean: 18.87 ± 3.87, *p* = 0.001).

### 2.2. Energy Availability Questionnaire (m-DEAQ)

The participants completed a Hebrew-language version of the modified dance-specific energy availability questionnaire (m-DEAQ) (adjusted for sex), which was modified for acrobatic gymnasts. The DEAQ [18]—created and validated for male and female dancers [18], whose characteristics are similar to acrobatic gymnasts—is based on several validated questionnaires for athletes, as described by Keay et al. [18,19,20]. The m-DEAQ sections cover common sporting and health characteristics associated with LEA and REDs, including (1) self-reported anthropometric characteristics (age, height, weight, etc.), (2) questions regarding training (frequency, duration, and intensity), (3) perceptions and beliefs regarding body weight and eating habits, and (4) training-related psychological and medical conditions. The Durham team created the data analysis methodology and a novel quantitative rating for REDs [18]. Based on the calculations, each participant received a REDs risk score (ranging from −6 to 11). A score lower than 0 indicates an increased risk of REDs. The score is different than the traffic light of the IOC team [2].

### 2.3. Anthropometrics and Body Composition Assessment

Weight and height were recorded, and the body mass index (BMI) percentile was calculated by a single technician using calibrated scales and stadiometers (H150-01-5, Shekel, Israel). The anthropometric data collected by the technician were used for a formal analysis. Body composition was assessed by dual-energy X-ray absorptiometry (DXA) scans performed by an experienced technician using a Discovery A densitometer (Hologic, Bedford, MA, USA). These included acquisitions of the lumbar spine, forearm (radius: 1/3), and total body (without the head). A standard automated report was generated using the manufacturer’s built-in software (version 13.6.0.7) [21]. The whole-body (without the head) composition indices documented were hip and spine BMD (g/cm^2^), total bone mineral content (BMC) (g), total lean body mass (LBM) (g), lean mass of the dominant arm (g), fat mass (g), fat percentage (%), and fat-free mass index (FFMI—total LBM + BMC divided by height squared [kg/m^2^]). The output included a Z-score using Hologic’s built-in database of children, which includes adjustments for age, sex, and ethnicity. A structured correction of the Z-score for height was performed to prevent bias due to low stature or developmental delay [22]. A nuclear medicine physician (author M.W.S.) reviewed and interpreted all reports according to accepted guidelines by the International Society for Clinical Densitometry (ISCD).

### 2.4. Energy Balance Assessment

The research team’s dietician (author N.R.) evaluated energy intake (EI) and nutrient consumption from 3-day food logs; the food log always recorded two weekdays and one day on the weekend [23]. The specific length of the food log was chosen for maximal compliance and to reduce missing data, considering the participants’ ages and daily activities. Participants took photographs of their meals with a measurement scale in view to assure accuracy. Nutrient data were coded and analyzed using NutRatio [24]. The software is based on a combination of three reliable databases, including the USDA (United States Department of Agriculture) database (SR-28 version, updated as of August 2018), data from the Ministry of Health as of July 2017, and information from food label data as of May 2019. Daily dietary analyses included energy, calcium, carbohydrates, dietary fibers, fats, fluids, folate, iron, phosphorus, proteins, zinc, and vitamins A, B-6, B-12, C, D, E, and K. For micronutrient level assessments, we used the recommended dietary allowance (RDA) as set forth by the Food and Nutrition Institute of Medicine [25]. Exercise energy expenditure (EEE) was calculated using the 2011 Compendium of Physical Activities [26]. This was adjusted to remove calories contributed by the resting metabolic rate (RMR) for the reported duration of exercise [15]. The RMR was estimated using the Cunningham equation of RMR = 500 + (22×FFM) [27]. Energy availability (EA) was calculated as the daily energy intake (EI) minus EEE, divided by the fat-free mass (FFM) derived from the DXA scans [2]. An EA adequate for growth and development is ≥45 kcal/kg FFM/Day, a reduced EA is 30–45 kcal/kg FFM/Day, and LEA is ≤30 kcal/kg FFM/Day. All athlete participants were offered web-based virtual meetings with the study team (usually with the dietitian), focusing on their personal data collected during the study and offering specific nutritional guidance. An athlete was referred to a medical doctor if further assistance was warranted.

### 2.5. Activity Tracker

Each gymnast was given a Vivosmart 4 activity tracker (Garmin, Olathe, KS, USA) and was instructed to wear it during the entire duration of their three days of food logging. The data gathered by this watch-type device included steps per day, hours of sleep, minutes of activity (light/intense), floors climbed, and average daily heart rate. The information was gathered blinded to the investigator (author O.B.).

### 2.6. Statistical Analysis

Anthropometric data and DXA test results were presented through descriptive statistics (mean, median, and standard deviation). We also performed several subgroup analyses at different levels: competition level (international vs. national), position on the team (top vs. base—a “middle” position can exist; however, due to position demand similarities, for our purposes, these individuals were included in the “top” group), and sex (male vs. female). The m-DEAQ was analyzed as a continuous variable. Comparisons of means were performed using an independent-samples *T*-test or the Mann–Whitney U test, which is based on assumptions testing (normal distribution, etc.). Similar analyses were performed on the continuous variables from the DXA scans. All analyses were performed using IBM SPSS Statistics (version 25).

## 3. Results

There were no significant differences between sexes in any of the anthropometric indices (Table 2). One-third of the males (n = 2) and a third of the female acrobats (n = 4) had a negative BMI percentile (−1 < Z-score < 0). Another third of the females (n = 4) had a negative Z-score with an SD greater than 1 (Z-score < −1).

**Figure 1:**
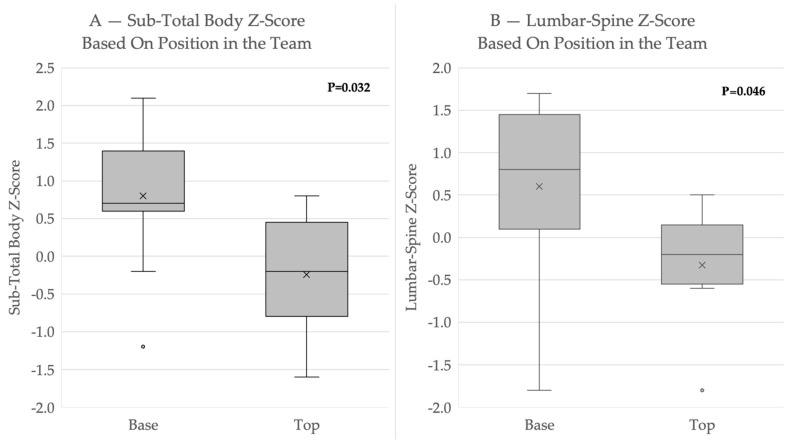

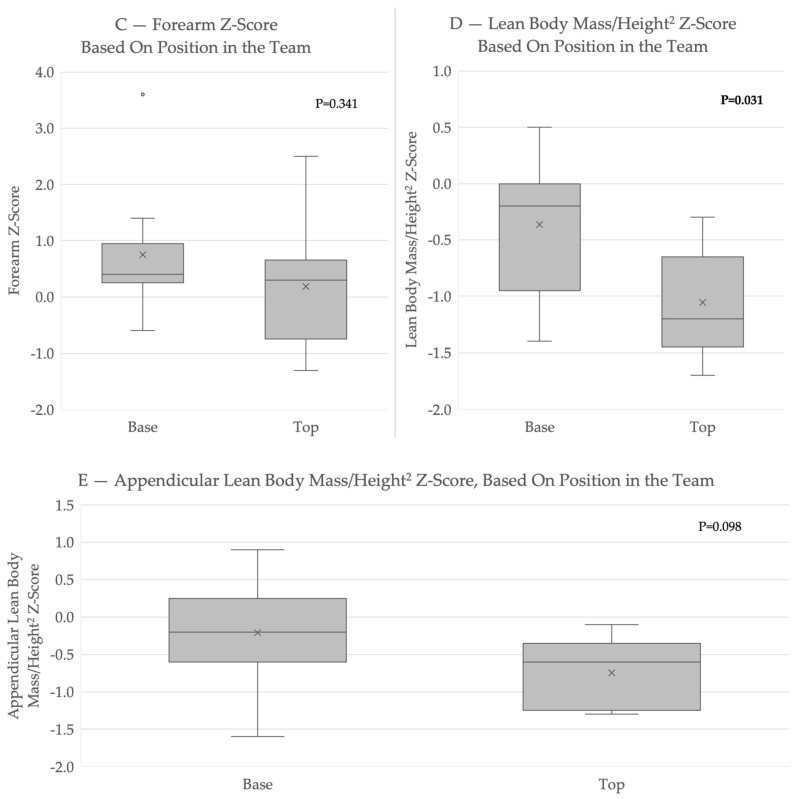
Z-score comparison by position in the team (base vs. top). Significant differences between the base and the top are marked in bold (*p* < 0.05).

All athletes had a BMD within the normal range for healthy adolescents at all measured sites, with a Z-score greater than −2.0. Three female acrobats had low BMDs for athletes (Z-score < −1) [7,20]. The BMD Z-scores of the gymnasts were lower for top-position athletes compared to those in base positions (−0.24 ± 0.93 vs. 0.80 ± 0.91, *p* = 0.032) (Figure 1 and Table A1).

The acrobats’ sub-total body Z-scores strongly correlated with their lumbar spine Z-scores (r = 0.879) and their forearm Z-scores (r = 0.748). In a subgroup analysis for each position, the correlations were weaker in the base-position group for the lumbar spine (r = 0.785) and the forearm (r = 0.7). In the top-position group, correlations were stronger between the sub-total body and the forearm (r = 0.882) than for the lumbar spine (r = 0.811).

**Figure 2:**
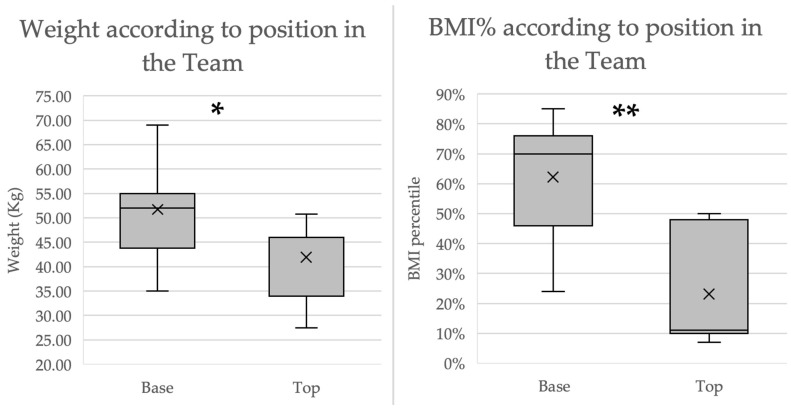
Position’s relation with weight and BMI percentile. Top-positioned acrobats had significantly lower weight (**left figure**) and BMI %tile (**right figure**) values compared to base-position acrobats. * Significant difference discovered. * *p* = 0.041; ** *p* < 0.001.

On their teams, 11 of the acrobatic gymnasts were base-position athletes, while the other seven were top-position atheletes. All male participants were base-position acrobats. In comparison to their top-position teammates, base-position gymnasts (males and females combined) were in a higher BMI percentile (62.18 ± 19.5 vs. 23.1 ± 18.6, *p* > 0.001). The DXA scans showed no significant difference in fat percentage (23.1 ± 6 vs. 20.5 ± 2.3, *p* = 0.223). An analysis of female participants based on their position revealed that base athletes were in a significantly higher BMI percentile than top acrobats (65.6 ± 19.8 vs. 23.1 ± 18.6, *p* = 0.0035). The DXA-measured body fat percentage for top-position gymnasts was also lower (20.49 ± 2.31% vs. 26.32 ± 4.39%) (*p* = 0.013) (Figure 2).

The mean EA for females was 27 ± 14.3 kcal/kg FFM/day, which was lower than the EA for males (39.6 ± 18.5 kcal/kg FFM/day). Two-thirds of the athletes (eight female and four male) had LEA. There were no significant differences based on position or competition level.

**Figure 3:**
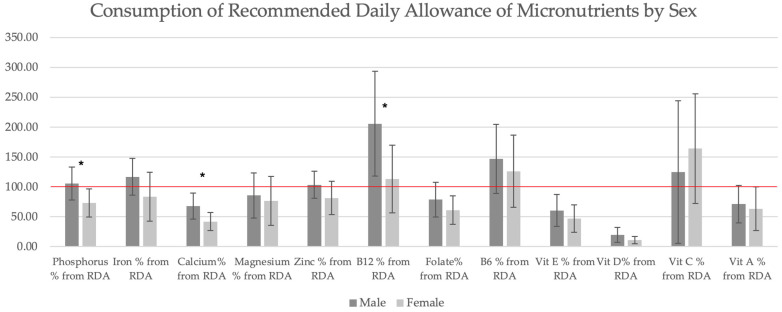
Consumption of the recommended daily allowance of micronutrients. Consumption of the recommended daily allowance (RDA) of micronutrients shown in percentages by sex. A red line situated at 100% of the RDA. * Significant difference as measured by the Mann–Whitney test, *p* < 0.05. RDA—recommended dietary allowance.

The consumption of micronutrients by sex is displayed in Figure 3. The overall micronutrient consumption for males was higher than for females; iron consumption was insignificantly higher (10.47 vs. 8.73 mg/day). Males consumed significantly more calcium (883 vs. 544 mg/day), phosphorus (1318 vs. 914 mg/day), and vitamin B12 (4.1 vs. 2.3 mg/day). Vitamin C consumption was higher for females than males. For all participants, calcium and vitamin D consumption was lower than the RDA. The macronutrient data and EA calculated are displayed in Table 3.

The fitness tracker data showed that the athletes slept a mean of 8.1 ± 0.7 h a night. The average steps per day was 9002 for males and 9602 for females. Four females reported that trainers gave them dietary recommendations. Eight females and one male were told at some point in their careers to lose weight. Members of the international team reported more team weigh-ins than the national team (*p* = 0.018). The gymnasts’ principal nutritional counseling resources were family and friends (46.1%) and coaches (23.1%), while only a single acrobat had consulted a dietitian.

**Figure 4:**
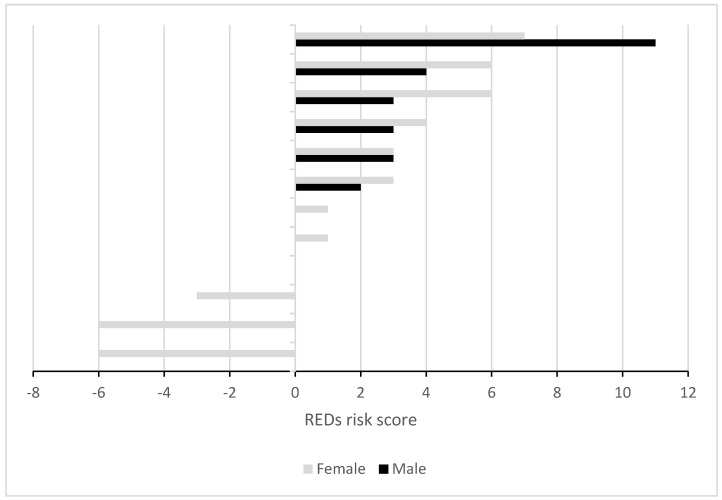
Calculated REDs risk score displayed by sex. There were 6 male participants, shown in the top portion of the figure next to the females; at the bottom of the figure, only females are shown. One female had a score of 0. REDs—relative energy deficiency in sport.

The REDs score distribution for all participants is displayed in Figure 4. Scores for females were lower than those for males (1.33 ± 4.44 vs. 4.33 ± 3.32, *p* > 0.05). The international athletes had lower REDs scores compared to the national ones. Only among top-positioned acrobats were there negative scores.

## 4. Discussion

This is the first single-center prospective study to evaluate body composition, bone health, energy balance, and eating and training habits in adolescent acrobats. The main finding was that in comparison to base-position acrobatic gymnasts, top-position athletes have a lower BMD in all regions except for the hands (sub-total body, lumbar spine, and lean body/height). The comparison was based on Z-scores to adjust for sex, age, and ethnicity. Among the top-position acrobats, three had BMD Z-scores < −1, which is considered low for athletes [7,20] and puts them at an increased risk of bone fracture and a decrease in peak bone mass [28]. Interestingly, the forearm Z-score did not show a significant difference between the two positions. Therefore, weight-bearing activities demonstrated bone health improvement in the acrobatic gymnasts and a lower-than-expected BMD for top-position acrobats. A possible etiology might be due to the caloric restriction of top-position acrobats combined with their increased training load. Base-position acrobats engage in more high-impact training such as jumping and standing routines and are also called upon to bear the weight of top-position acrobats, who engage in lower-impact training. Top-position acrobats must carry their body weight on their hands for long periods, with their forearms being subjected to higher-than-normal strain, which increases their BMD. The low BMD of the top-position acrobats was consistent with findings in non-weight-bearing sports such as synchronized swimming [29]. On the contrary, the observation of a normal BMD only in specific high-strain areas (forearms) is similar to the effect seen in gymnastic categories [29]. The top-positioned athletes, while they perform relatively low-impact training, are often encouraged to be as light as possible and decrease their food consumption, increasing their risk of LEA. This condition is similar to other sports which also encourage their athletes to have a lean physique, whether for judges’ points or for better performance (horse riding and cycling), endangering their athletes by causing a higher risk of LEA and REDs. Interestingly, a sex difference was not observed, possibly due to the small sample size. The assessment of BMD in this research has portrayed the unique features of this sport. Each position has its own characteristics, which makes it difficult to compare this sport to other sports, whether weight-bearing or not.

Another important finding is the high risk of REDs based on the m-DEAQ scores found for top-position and female acrobats, rendering them higher-risk for morbidity (e.g., injuries, as in other esthetic sports [30] and impaired performance. Low weight demands for top-position acrobats could drive them to LEA and, in turn, REDs symptoms and subsequently to low BMD. Top-position acrobats are also younger and have a lower body fat percentage. To achieve optimal performance, the recommended EA for teen athletes is 45 kcal/kg FFM/day [1], a goal only five acrobats achieved. As expected, the EA values for males and females differed, yet both were below the recommended EA for athletes [1,18]. While the males’ mean EA was slightly decreased compared to the recommendations (mean: 39.6 ± 18.5 kcal/kg FFM/day), the female mean EA was much lower (mean: 27 ± 14.3 kcal/kg FFM/day). A staggering 66% of the female acrobats in this study had LEA. Interestingly, the international-level acrobats, who trained significantly more hours per week, did not have a higher LEA rate, REDs, or showed any difference in BMD. This can be explained by genetic predisposition, meaning that the international-level athletes were genetically more “suitable” to the sport in terms of body build, as described by Hamilton [31], who found fewer eating disorders in more elite dancers. Another explanation might be that the international athletes are educated and know that they must practice healthy habits in order to prevent frequent injuries, as suggested by Keay et al. for professional dancers [32].

Our results differ from Silva et al., who reported REDs symptoms for acrobats in Portugal [5], although their calculated EA was 114–121% higher than that calculated in our study. This difference could occur for several reasons: different populations in terms of ethnic origin, small numbers of subjects, different methods of measuring EA, and body composition measurement techniques.

It is alarming that 66% of the female acrobats reported that they were told by their coaches to lose weight. Most of them (87%) reported following a diet intended to help them lose or maintain weight. Only one of the gymnasts had ever consulted a professional dietician. It is recommended that athletes, especially those participating in “high-risk” sports in terms of REDs, should receive professional nutrition guidance to balance their nutritional needs and improve their performance and long-term health outcomes [33]. As indicated above, another source of stress is the self-reported feeling of needing to lose weight after engaging with social media [34]. Similar stress sources were reported for dancers [32]. While not subject to coaching team control, awareness of the phenomena and open communication between athletes, parents, and coaches about social media’s impacts is important and could mitigate their effects.

As opposed to the traffic light method currently in use [20], the m-DEAQ questionnaire [18] was designed to allow for, for the first time, a quantitative assessment of the risk of REDs, which showed that the acrobats with the lowest scores (highest risk) were those with the lowest EA. This unique tool could possibly assist physicians and trainers as a screening tool for REDs assessments in athletes and in future studies.

## 5. Limitations

Firstly, while our results are novel and interesting, due to the small sample size, we were limited in our analyses, including correlations and regression models. New barriers to recruiting patients to in-hospital studies during and after the COVID-19 pandemic have become more prominent, and we intend to enroll more acrobats in future field/out-patient studies.

We did not assess pubertal maturation in person as we felt this might limit participation. For example, in males, morning erections are an important sign of proper energy availability. However, we believed that inquiring about this matter with boys under 16 years of age would be inappropriate. Half of the females we studied reported being pre-menarcheal. However, due to their ages (all below 15), it is too early to determine whether they have delayed menarche due to LEA as only primary amenorrhea is considered an indicator of REDs.

In order to calculate the required sample size, a comprehensive literature survey was conducted to learn about feasible endpoints. No research studies on the acrobatics population or on a similar population using DXA were discovered. Research protocols for athletes with similar characteristics vary significantly; thus, sample size was difficult to estimate.

In order to not interfere with their training, some of the athletes removed their fitness tracker watches during practices. Accordingly, for further studies, other methods of activity tracking during practice should be implemented (the use of a heart monitor and an activity tracker simultaneously).

## 6. Conclusions

In this prospective study evaluating adolescent acrobatic gymnasts, while BMD parameters were within the normal range for a pediatric population, three acrobats (16%) had lower BMD than expected for athletes [7]. Position-based differences were found for BMD parameters. The BMD Z-scores for the lumbar spine and total body were significantly lower for top-position gymnasts compared to their base-position counterparts, while scores for the arms were similar between groups. Another alarming finding is the high risk of REDs found for top-position and female acrobats. These findings suggest that top-position athletes might benefit from the addition of high-impact activities (such as jumping) to potentially increase their total body BMD. There is also room to focus on balanced and sport–gender–position-specific nutrition, preferably via professional resources. Larger long-term, comprehensive studies that include more male acrobats are needed to identify the unique aspects of this increasingly popular sport.

## Figures and Tables

**Table 1:** Health and performance aspects impacted by REDs [2]. Lower EA correlates to worse health and performance outcomes in each aspect. There are more aspects impacted, as noted in Mountjoy et al., 2023 [2]. REDs—relative energy deficiency in sports; EA—energy availability.

REDs Health Conceptual Model	REDs Performance Conceptual Model
Impaired reproductive function	Decreased endurance performance
Impaired bone health	Decreased training response
Impaired growth and development	Decreased recovery
Impaired energy metabolism/regulation	Decreased motivation
Mental health issues	Decreased power performance
Impaired glucose and lipid metabolism	Decreased cognitive performance/skill
Impaired gastrointestinal function	Decreased muscle strength

**Table 2:** Acrobats’ anthropometric data. Data are shown as mean ± SD values or percentages (%). The *p*-values displayed were calculated using a simple Student’s *T*-test for unpaired samples. * The resting metabolic rate (RMR) was calculated using Cunningham’s equation [27]. BMI—body mass index; DXA—dual-energy X-ray absorptiometry; RMR—resting metabolic rate.

	Female	Male	*p*-Value
Number of Acrobats	12 (66%)	6 (33%)	
Height (cm)	155.7 ± 10.9	157.2 ± 9.5	0.77
Height (%tile)	36.6 ± 35.4	31.1 ± 18.6	0.68
Weight (kg)	46.3 ± 8.2	50.9 ± 13.2	0.46
Weight (%tile)	38.2 ± 24.6	45.7 ± 19	0.524
BMI (kg/m^2^)	19 ± 2.4	20.2 ± 2.9	0.379
BMI (%tile)	40.8 ± 28.4	59.3 ± 20.6	0.178
BMI (Z-score)	−0.32 ± 0.8	0.25 ± 0.56	0.169
Body fat by DXA (%)	22.9 ± 4.3	20.3 ± 6.1	0.385
Fat-free mass (%)	77 ± 4.3	79.6 ± 6.1	0.385
Fat-free mass (kg)	35.5 ± 5.4	40.9 ± 12.7	0.357
RMR * (kcal/day)	1280.9 ± 119.1	1399.9 ± 279.5	0.357

**Table 3:** Energy and macronutrient comparisons calculated for the sexes (mean ± SD). EI—energy intake; EEE—exercise energy expenditure; EA—energy availability.

	Female	Male	*p*-Value
Number (%)	12 (66%)	6 (33%)	
EI (kcal/day)	1412 ± 445	2053 ± 375	0.008
EEE (kcal/day)	644 ± 148	772 ± 419	0.350
EA (kcal/kg FFM/day)	27 ± 14.3	39.6 ± 18.5	0.129
Energy (kcal/kg/day)	31.9 ± 12.7	42.6 ± 12.1	0.118
Carbohydrates (gr/day)	179 ± 54	244 ± 53	0.028
Carbohydrates (gr/kg/day)	4 ± 1.5	5 ± 1.5	0.201
Protein (gr/day)	61.3 ± 25.1	92.3 ± 25	0.025
Protein (gr/kg/day)	1.4 ± 0.7	1.9 ± 0.8	0.165
Fat (gr/day)	51 ± 18.7	78.8 ± 9.9	0.004
Fats (gr/kg/day)	31.9 ± 4.1	34.9 ± 2.7	0.132

## Data Availability

Data will be available upon reasonable request from the corresponding author.

## Abbreviations

BMC—bone mineral content; BMD—bone mineral density; BMI—body mass index; DXA—dual-energy x-ray absorptiometry scan; EA—energy availability; EEE—exercise energy expenditure; EI—energy intake; FFM—fat-free mass; FFMI—fat-free mass index; IOC—International Olympic Committee; ISCD—International Society for Clinical Densitometry; LBM—lean body mass; LEA—low energy availability; LS—lumbar spine; m-DEAQ—modified dance-specific energy availability questionnaire; RDA—recommended dietary allowance; REDs—relative energy deficiency in sports; RMR—resting metabolic rate; USDA—United States Department of Agriculture.

## Appendix A

**Table A1:** Bone mineral density in different body areas by acrobatic position in the team. The values in the table are shown as mean ± SD values. Significant differences are highlighted in bold (BMD—bone mineral density).

		Base	Upper	*p*-Value
	Number (%)	11 (61%)	7 (39%)	
Sub-total Body	BMD (g·cm^2^)	0.13 ± 0.97	0.08 ± 0.88	0.169
	Z-score	0.80 ± 0.90	−0.24 ± 0.93	**0.032**
Lumber Spine (L1–L4)	BMD (g·cm^2^)	0.92 ± 0.21	0.87 ± 0.11	0.529
	Z-score	0.60 ± 1.04	−0.32 ± 0.76	**0.046**
Forearm	BMD (g·cm^2^)	0.66 ± 0.08	0.64 ± 0.07	0.555
	Z-score	0.74 ± 1.09	0.18 ± 1.30	0.341
Lean/Height^2^	Kg/m^2^	14.92 ± 2.12	13.07 ± 0.72	0.042
	Z-score	−0.36 ± 0.63	−1.06 ± 0.57	0.031
Appendicular Lean/Height^2^	Kg/m^2^	6.76 ± 1.21	5.76 ± 0.38	0.050
	Z-score	−0.21 ± 0.68	−0.74 ± 0.52	0.098
